# Management Aspects of Medical Therapy in Graves Disease

**DOI:** 10.1016/j.eprac.2024.12.012

**Published:** 2024-12-17

**Authors:** Rutu Shah, Samantha E. Adamson, Sina Jasim

**Affiliations:** Division of Endocrinology, Metabolism and Lipid Research, Washington University in St. Louis, School of Medicine, St. Louis, Missouri

**Keywords:** Graves disease, antithyroid drugs, thyroid function tests, hyperthyroidism

## Abstract

**Objective:**

Graves disease (GD) is the most common cause of hyperthyroidism. Treatment options include antithyroid drugs (ATDs), radioactive iodine, and surgery. In this review, we focus on the medical aspects of managing GD.

**Methods:**

The authors conducted a literature review of PubMed to include studies and review articles on GD management, ATDs, long-term safety of antithyroid drugs, hyperthyroidism in pregnancy, Graves ophthalmopathy, and special circumstances related to hyperthyroidism.

**Results:**

In adjunction to ATDs, medical management for GD also includes beta-blockers, glucocorticoids, and iodine containing agents. ATDs are currently the preferred option for initial management of GD, reflecting a shift in practice observed in the United States over the past 2 decades. ATDs in appropriate doses are well-tolerated and safe when used for longer duration, during pregnancy, and other circumstances discussed in this article. Routine thyroid function tests are important for monitoring. Thyrotropin receptor antibody plays an essential role in determining duration of treatment and assessing the likelihood of recurrence.

**Conclusion:**

Medical management of GD with antithyroid drug is safe and effective. Long-term use beyond 24 months in patients with elevated thyrotropin receptor antibody is a reasonable alternative option to surgery and radioactive iodine due to higher rates of remission.

## Introduction

Graves disease (GD) is the most common cause of hyperthyroidism. It is an autoimmune disorder with systemic manifestations that primarily affect thyroid gland, leading to hyperthyroidism and diffuse goiter, ophthalmopathy, and, rarely, dermopathy.

The overall prevalence of hyperthyroidism in the United States is 1.2% with an incidence of 20/100,000 to 50/100,000, typically affecting the age group between 20 and 50 years. GD accounts for 60% to 80% of these cases, more common in women than in men with lifetime risk of 3% and 0.5% in women and men, respectively.^1^

GD occurs more commonly in patients with a positive family history and genetic predisposition, precipitated by environmental factors such as stress, smoking, infection, iodine exposure, postpartum status, and certain medications (Table 1).^2^

Current treatment options are all effective and include medical therapy with antithyroid drugs (ATD), surgical therapy, and radioactive iodine (RAI), each of which is associated with potentially serious side effects. When guiding patients through shared decision making to best treatment option, physicians should review both immediate risks and benefits of therapy, as well as long-term effects.

ATD has been the first-line treatment for half a century worldwide, but RAI therapy was used more often in treating GD in United States. However, a shift of treatment preference was noticed over the past few years, with a sharp decline in the use of RAI as initial therapy for GD from 69% in 1990 to 11.1% in 2023^3^ and wider use of ATD in the United States based on a survey of clinical endocrinologists.

The medical management of GD was described in detail in previous review of this journal 30 years ago.^4^ Updates on diagnostic assays, duration of ATD use, monitoring and predicting remission, as well as additional options for treating eye disease have been developed since.

This review focuses on the medical management of Graves hyperthyroidism and long-term safety of ATD.

## Pathogenesis of GD

The pathogenesis of GD involves a complex interaction between genetic and environmental factors resulting in loss of self-tolerance to thyroid antigens.^5^ B lymphocytes, stimulated by T lymphocytes (sensitized by antigen), lead to synthesis of thyroid-stimulating immunoglobulin (TSI) within the thyroid tissue. TSI binds with thyroid-stimulating hormone (TSH) receptor on the thyroid cell membrane and simulates the action of TSH which leads to thyroid hormone synthesis and thyroid gland growth, causing hyperthyroidism and thyromegaly.

Thyroid eye disease (TED) is caused by inflammation, cellular proliferation, and increased growth of extraocular muscles and retro-orbital connective and adipose tissues due to the effect of thyroid-stimulating antibodies and released cytokines leading to excess hydrophilic glycosaminoglycans and retro-orbital fat growth, and subsequent proptosis, diplopia, congestion, and periorbital edema.^6^ Insulin-like growth factor (IGF)-1 enhances the action of thyrotropin and immunoglobulins that activate IGF-1 receptor signaling and is detected in patients with GD.^7^ The pathogenesis of other rare manifestations of GD like pretibial myxedema and thyroid acropachy is poorly understood.

## Diagnosis of GD

Awareness of common and atypical presentations of GD is necessary as failure to diagnose GD in a timely manner may lead to higher morbidity and mortality. Most patients with GD present with classic signs and symptoms of hyperthyroidism, but they can also present with TED in about 40%^8^ of cases and dermopathy in 5% to 15% of patients.^9^ Thymic enlargement has also been reported.^10^ Clinical presentation depends on the age of onset, severity, and duration of hyperthyroidism.

Common presentations such as heat intolerance, sweating, fatigue, weight loss, palpitation, tremors, anxiety, nervousness, as well as eye symptoms and palpable goiter are often seen in younger population. In older patients, symptoms are usually subtle with fatigue, weight loss, and new onset atrial fibrillation. Atypical presentation of hyperthyroidism in older adults is also referred as apathetic thyrotoxicosis.

The initial test for diagnosing hyperthyroidism is TSH. If TSH is suppressed, free T4 (FT4) and free T3 (FT3) should be measured. Suppressed TSH with high FT4 or FT3 or both confirms the diagnosis of hyperthyroidism. In subclinical hyperthyroidism, TSH is suppressed, with normal FT4 and FT3. Total T4 and total T3 can be ordered if free hormone assays are unavailable. A FT3/FT4 ratio >0.3 or total T3/T4 ratio of >20 ng/μg may suggest GD.^11^

Further testing with TSH/thyrotropin receptor antibodies (TRAb) can be helpful in differentiating GD from other causes of hyperthyroidism. TRAb is more cost effective than images, and if positive, can confirm the diagnosis of GD. Measurement of TRAb level is a standard method for diagnosing, monitoring therapy, and prediction of remission or relapse in patients with GD.^12^ There are 2 available methods for detecting antibodies against TSH receptors: thyrotropin-binding inhibiting immunoglobulin (TBI/thyrotropin-binding inhibitory immunoglobulin) assay and the functional TSI bioassay. Although TSI is a subtype of TRAb, TBI assays are more commonly referred to as TRAb. TBI assays have evolved over time and the current third-generation binding assays are automated and have improved sensitivity and specificity of up to 97% and 99% for diagnosing GD.^13^ However, these are competitive assays and do not differentiate between stimulating, inhibiting, or neutral Abs. TSI, on the other hand, is a cell-based bioassay that specifically detects stimulating immunoglobulins and, when compared with TBI, is more sensitive.^14^ TRAb measurement can particularly be useful in certain clinical scenarios (Box).^1^ Although there is no consensus on the preferred type of immunoglobulin, following TSI has not shown a significant advantage over TRAb in predicting and managing Graves ophthalmopathy specifically.^15^ In clinical practice, competitive immunoassays are faster, automated, and more readily available, and therefore are used more commonly.^16^

Additional biochemical findings in hyperthyroidism are microcytic anemia, thrombocytopenia, bilirubinemia, high transaminases, hypercalcemia, high alkaline phosphatase, low-density lipoprotein and high-density lipoprotein cholesterol. Images are not routinely necessary for diagnosing GD but can be helpful. When thyroid antibodies are negative, imaging such as neck ultrasound to show hypervascularity and use of elastography in GD may help differentiate GD from other causes of hyperthyroidism.^17^ Radionuclide thyroid scan can show increased and diffuse thyroid uptake consistent with GD but is not necessary to perform routinely if biochemical tests are confirmatory. Other images such as computed tomography or magnetic resonance imaging of orbits can be performed to diagnose TED.

## Medical Management of GD

### Initial Management

Initial management of hyperthyroidism due to GD often occurs while awaiting confirmatory testing. The goals of initial management are to mitigate symptoms of hyperthyroidism using beta-adrenergic receptor antagonists and normalize thyroid hormone levels with ATDs.

### Beta-Adrenergic Receptor Antagonists

Beta-adrenergic receptor antagonists ameliorate symptoms of hyperthyroidism that are caused by increased adrenergic tone like tremors, palpitations, tachycardia, heat intolerance, and anxiety. They should be initiated as soon as the diagnosis of hyperthyroidism is made. All beta-blockers are equally effective in relieving such symptoms. Propranolol is nonselective beta-blockade that has been historically used to control adrenergic symptoms because it blocks the peripheral conversion of FT4 to FT3.^18,19^ However, its short duration of action and need for multiple doses a day has given way to preferred use of long-acting beta-1 receptor selective blockers like atenolol and metoprolol with once-a-day dosing (Table 2).^20–22^ In the setting of severe thyrotoxicosis or thyroid storm in patients with hemodynamic instability, esmolol drip in the intensive care setting can be considered. Common side effects of beta-blockers are described in Table 2.

As thyroid hormone levels normalize and symptoms resolve, beta-blockers can be tapered and ultimately discontinued. In patients who do not tolerate or are not candidates for beta-adrenergic blockade, calcium-channel blockers like verapamil and diltiazem have been shown to affect rate control.^20^

### ATDs—Thionamides

**Fig. 1.**
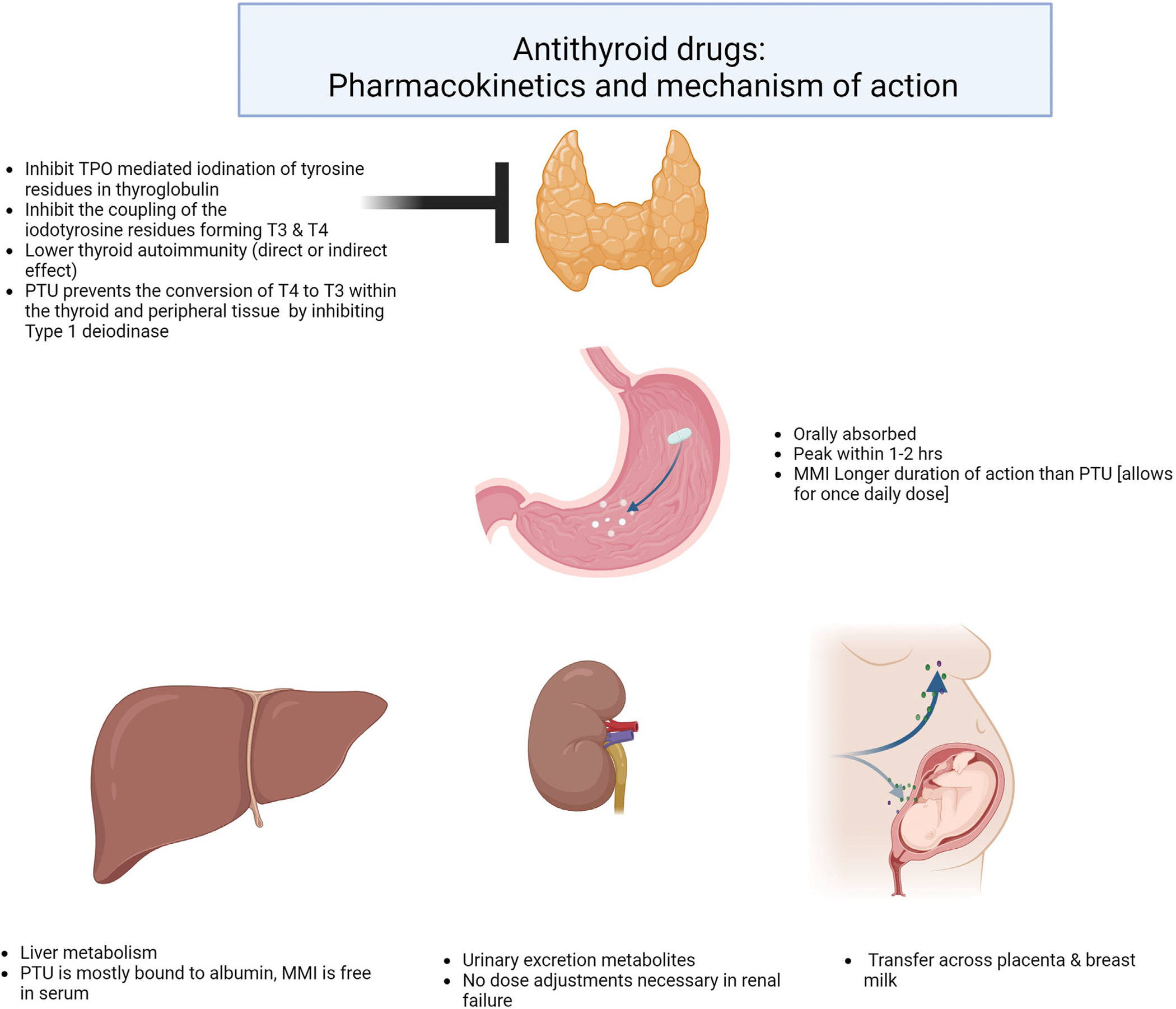
Antithyroid drugs: pharmacokinetics and mechanism of action. *MMI* = methimazole; *PTU* = propylthiouracil; *T4* = thyroxine; *TPO* = thyroid peroxidase; *T3* = triiodothyronine.

Methimazole (MMI) and propylthiouracil (PTU) are the only 2 ATDs available in the United States. Outside the United States, carbimazole, which rapidly metabolizes to MMI, is also available. Thionamides inhibit thyroid peroxidase (TPO)–mediated iodination of thyroglobulin in the thyroid gland and block the synthesis of T4 and T3. PTU also blocks peripheral conversion of T4 to T3. Some data suggest that ATD can affect thyroid autoimmunity given the decline in thyroid-stimulating antibody titer with ATD use and possible remission of GD on medical therapy alone.^23^ The mechanism of action and pharmacokinetics of ATD is described in Figure 1.

In nonpregnant patients, MMI is the drug of choice. MMI is preferred over PTU because of its better efficacy, safety, and longer duration of action allowing for once-a-day dosing regimen.^20,24,25^ The American Thyroid Association (ATA) recommends starting MMI at 10 to 30 mg daily to restore euthyroidism. Initial MMI dosing depends on the severity of hyperthyroidism^26^ and the degree of FT4 elevation. If FT4 level is 1 to 1.5 times the upper limit of normal, initial MMI dose of 5 to 10 mg daily is given; if level is 1.5 to 2 times the upper limit of normal, initial MMI dose of 10 to 20 mg daily is given; and if levels are 2 to 3 times the upper limit of normal, initial MMI dose of 30 to 40 mg daily is given. Split dosing of MMI may be more effective than a single daily dose when more rapid achievement of euthyroidism is needed.^20^ When requiring >30 mg/d, split dosing can help reduce gastrointestinal side effects. The equivalent PTU to MMI doses can be calculated using an average ratio of 15–20 to 1.^20^

Thyroid function should be monitored every 2 to 6 weeks and ATD dose should be titrated based on serum FT4 and FT3 levels as serum TSH levels can remain suppressed for several months.^20^ Once biochemical euthyroidism is achieved, the dose of MMI can be further titrated down to the lowest dose that is needed to maintain euthyroidism and follow-up intervals can be extended to 2 to 4 months. Overtreatment resulting in hypothyroidism should be avoided as it can exacerbate or provoke TED.^27^ Patients with newly diagnosed GD should be treated for 12 to 18 months with ATD according to the American and European practice guidelines^20,21^ with consideration for extended treatment duration in certain cases as discussed below.

Historically, the need for continued higher doses of MMI (>20 mg daily) was considered a reason to pursue definitive treatment such as RAI or surgery; however, studies have shown that long-term treatment with MMI is safe.^28,29^ ATDs are generally well-tolerated drugs but can have minor and major adverse effects. Minor side effects can occur in about 5% of patients and include gastrointestinal distress and pruritis. Major side effects including agranulocytosis and hepatotoxicity are rare and are dose-related^30^ for MMI and carbimazole. Agranulocytosis occurs in <0.5% of patients, typically in the first 3 months of treatment.^31,32^ It can present abruptly with fever, sore throat, or both. If confirmed, ATD should be discontinued permanently. The ATA recommends performing a complete blood count before starting ATD treatment.^20^ Hepatotoxicity is another potential serious side effect observed in <0.1% of patients. The pattern can be either cholestatic or hepatocellular and is generally more severe with PTU.^23^ European Medicines Agency issued a warning for acute pancreatitis with MMI in 2020 but the evidence for it has been conflicting.^33,34^

Baseline liver function testing before starting ATD is important since up to one third of patients with thyrotoxicosis have transaminitis. ATDs should be avoided if baseline transaminase levels are more than 5 times the upper limit of normal.^20^ There is no consensus about whether periodic monitoring of white counts or liver enzymes in predicting early adverse events. However, this practice is not routinely implemented in the United States due to the low incidence, abrupt onset, and lack of cost-effectiveness.^35^ It is important to educate patients on these adverse events at the time of starting these medications, including written information (Table 2).

The mainstay of medical management for GD is ATD and betablockers. However, additional agents listed below can occasionally be used to control hyperthyroidism.

### Bile Acid Sequestrants

Cholestyramine inhibits the enterohepatic circulation of T3 and T4. When used in combination with thionamides, it can help achieve euthyroidism more quickly in GD than thionamides alone.

### Iodine Therapy

Sodium ipodate and iopanoic acid inhibit peripheral conversion of T4 to T3. They serve as beneficial adjunct therapy with ATD, but not used alone. They are not available in the United States, instead potassium iodide drops, or saturated solution of potassium iodide, can be used in treating GD, thyroid storm, or preparation for surgery. At supraphysiologic doses, iodine reduces synthesis of new hormones (Wolff-Chaikoff effect). Its safety in pregnant women is controversial due to concerns for fetal goiter and hypothyroidism.

## Remission of GD on ATD

Remission from GD is defined as maintenance of biochemical euthyroidism for at least 1 year after stopping ATDs. Surgery and RAI therapy are considered more “permanent” treatment options for GD but cause lifelong hypothyroidism. Euthyroidism is likely to be achieved with ATD; however, withdrawal of ATD after the conventional 12 to 18 months of treatment results in recurrence of hyperthyroidism in 20% to 70% of patients.^20^

Features predicting response to ATD are small goiter, lesser degrees of thyrotoxicosis, and TRAb titers that are minimally elevated before ATD therapy or normalize on therapy. In a study, patients with serum TRAb values >4.4 IU/L had an 85% relapse rate and patients with TRAb concentrations between 0.9 and 4.4 IU/L experienced 53% relapse after MMI withdrawal. However, all those with TRAb concentrations <0.9 IU/L remained in remission.^36^ Apart from TRAb, other factors that can affect the outcome of ATD include FT4, age, thyroid volume, sex, family history, orbitopathy, smoking, and postpartum period. None of those factors alone are specific but they should be considered prior to stopping treatment (Table 3).^37–39^

Predictive scores for recurrence of GD, Graves Recurrent Events After Therapy (GREAT) for clinical markers (age, goiter size, serum FT4, and thyrotropin-binding inhibitory immunoglobulin) and GREAT+ for combination of clinical and genetic markers (protein tyrosine phosphatase nonreceptor type 22 and human leukocyte antigen alteration), were first described in 2016 by Vos et al^40^ during a 24-month follow-up period in 178 patients with GD treated with ATD for a year. Both scoring systems are divided into classes, I to III for GREAT and I+ to IV+ for GREAT+ based on certain markers to predict the risk of recurrence after discontinuation of ATD. GREAT class I (0–1 points) associated with a recurrence risk of 12% to 34%, class II (2–3 points) at 35% to 39%, and class III (4–6 points) with 53% to 74% recurrence risk. GREAT+ divides patients in 4 categories corresponding with the relapse rate of 4%, 21%, 49%, and 85%. The GREAT score was validated in 3 different cohorts with larger study populations,^41–43^ the need for external validation of GREAT+ score was recently proposed.^44^ A different study by Azizi et al^45^ found that the risk of hyperthyroidism recurrence was 56% in short-term compared with 17% in those treated long-term. Based on the risk factors associated with recurrence, a new risk scoring model was proposed. The predictors were similar to GREAT score with the addition of T3 levels and sex.

### Subsequent/Long-Term Management

Chronic ATD therapy is a reasonable alternative to surgery and RAI for GD in patients who fail to achieve remission after an initial course of ATDs.

Patients with GD treated with long-term ATDs showed a good and safe response. There was a 16% increase in sustained remission rate for each additional year of treatment beyond 24 months of ATD. Overall adverse effect rate was 19.1%, consisting mostly of rash, gastric intolerance, or arthralgia, with only 1.5% of patients experiencing severe reactions.^28^

In patients with diffuse toxic goiter who had recurrences of hyperthyroidism, long-term ATD treatment was superior to RAI when mood, cognition, cardiac function, and occurrence of thyroid dysfunction were compared.^46^ Moreover, compared with RAI, low-dose MMI in relapsed GD (after 12–24 months of initial treatment with MMI) was not only safe and effective, but had better outcomes for Graves orbitopathy.^47^

A study of 302 patients with GD randomized to receive MMI for 18 to 24 months (short-term) or 60 to 120 months (long-term). MMI was given as once daily at 20 to 30 mg daily for the first month. The dose was titrated at each study visit to achieve and maintain euthyroidism. Recurrence of hyperthyroidism was reported in 43% and 51% in the short-term group and in 8% and 15% in the long-term group within 12 and 48 months after MMI withdrawal, respectively. The MMI dose at the time of discontinuation was averaged at 5 mg daily.^48^

When discontinuation of ATD is considered, an assessment of likelihood of remission should be performed after 18 months of ATD treatment and ATD withdrawal can be considered only in patients who have serum TRAb level <0.9 IU/L.^49^ In patients with TRAb level >0.9 IU/L, ATD treatment should be continued. Long-term use of ATD is overall safe and effective as reported by multiple studies and euthyroidism could be maintained with small MMI dose (2.55.0 mg daily) during years of treatment in majority of patients.^50^ Very long-term beyond 15 years and even lifelong use of ATD is proved to be safe and effective.^29^

**Fig. 2.**
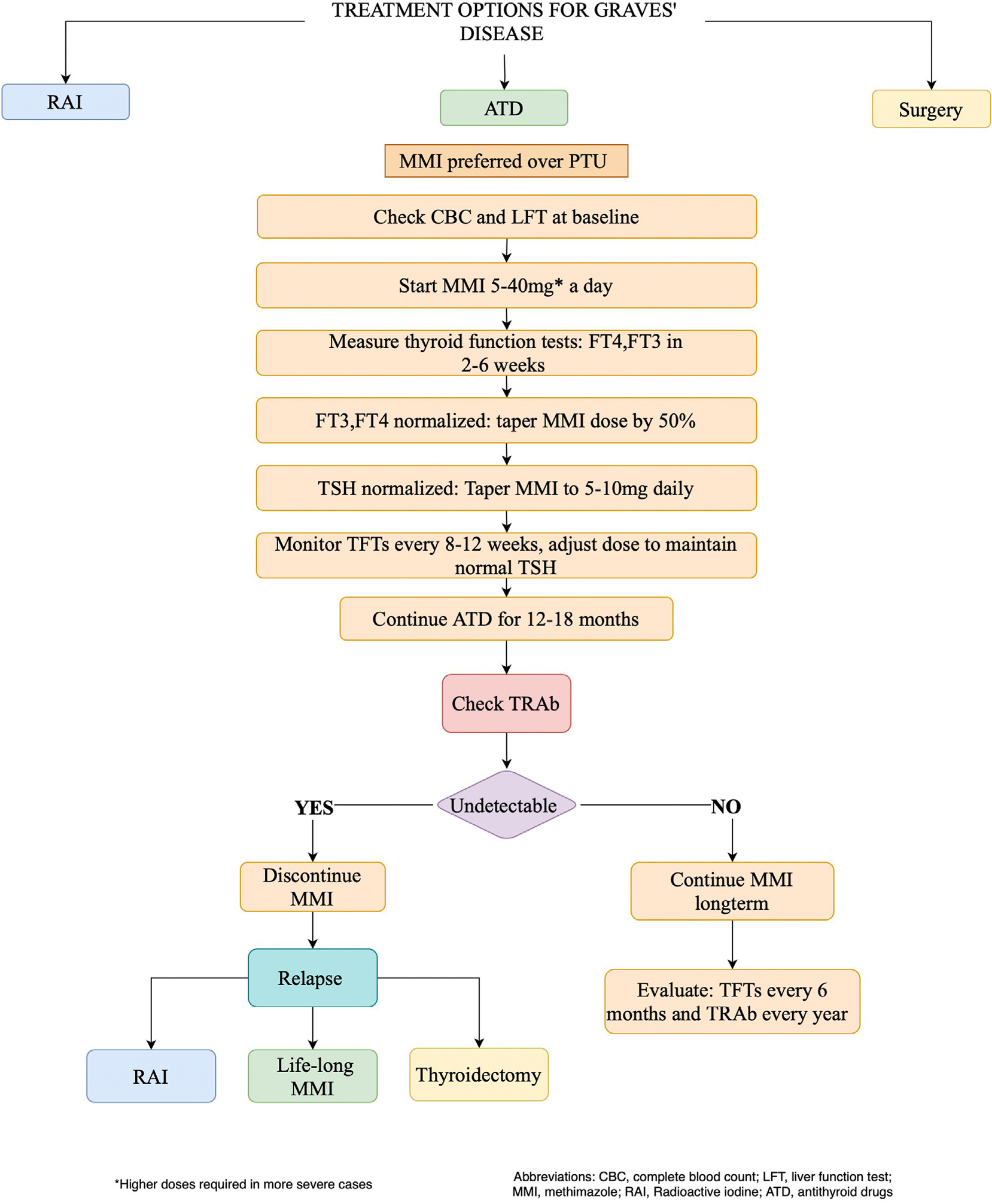
Proposed algorithm for initial and long-term medical management of Graves disease. *ATD* = antithyroid drug; *CBC* = complete blood count; *FT4* = free thyroxine; *FT3* = free triiodothyronine; *LFT* = liver function test; *MMI* = methimazole; *TFT* = thyroid function test; *TRAb* = thyrotropin receptor antibody; *TSH* = thyroid-stimulating hormone.

Proposed algorithm for initial and long-term medical management of GD is detailed in Figure 2.

## Special Circumstances

### Pregnancy and Lactation

Hyperthyroidism in pregnancy is uncommon and complicates approximately 0.1% to 0.4% of pregnancies with GD estimated to account for 85% to 95% of cases.^51^ Uncontrolled maternal hyperthyroidism poses a threat to the mother and fetus. Maternal hyperthyroidism is associated with increased incidence of gestational hypertension, pre-eclampsia, and gestational diabetes. Delivery and fetal-related complications include spontaneous miscarriages, premature delivery, and intrauterine growth restriction.^52,53^ Given the multitude of severe complications, overt hyperthyroidism should be treated. Subclinical hyperthyroidism in pregnancy should be closely monitored. Physiologic changes in thyroid function tests during pregnancy and interpretation of those test are detailed elsewhere.^54^

GD in pregnancy is treated with ATD. Both PTU and MMI can cross the placenta; therefore, the lowest therapeutic dose of ATD should be used. Birth defects are reported with both medications, although MMI and carbimazole have been linked with a rare embryopathy which includes a constellation of birth defects constituting developmental delay, aplasia cutis, choanal atresia, trachea-esophageal fistula, ventricular septal defects, omphalocele, and athelia/hypothelia with a distinctive facial phenotype. Use of PTU is linked with rare isolated congenital defects like development of cysts in head and neck and urogenital anomalies. However, the incidence of those effects with PTU is slightly lower than with MMI. Therefore, it is recommended to use PTU during organogenesis in the first trimester. Patients can then be transitioned to MMI in the beginning of second trimester to decrease the likelihood of hepatotoxicity with PTU.^55,56^ If the patient does not tolerate PTU, treatment with MMI is still preferred over no treatment at all. When switching between the 2 drugs, a ratio of 1:20 of MMI to PTU can be used as the initial conversion factor.

Beta-blockers can be used in pregnancy for symptomatic control and should be discontinued as soon as euthyroid state is achieved. They carry a risk of intrauterine growth restriction, fetal bradycardia, and neonatal hypoglycemia. Preferred agents include propranolol but in patients who prefer once-a-day dosing, metoprolol can be used.

During monitoring, thyroid function tests should be checked every 2 to 6 weeks aiming for serum free T4 levels at upper limit of reference range for nonpregnant women or total T3 levels at 1.5 times upper limit of range for nonpregnant women. TSH lags by weeks and is not a reliable marker. Once goal is achieved, dose can either be maintained with thyroid tests checked every 4 to 6 weeks or cut by 50% and monitoring labs repeated at shorter intervals of every 2 to 4 weeks.^57^ TRAb levels fall during pregnancy and ATD can be discontinued in about one third of patients after 30 to 34 weeks of gestation. Fetal hypothyroidism is an indication to decrease or stop ATD.^58^ ATDs also secreted in breast milk at low levels, but MMI dose up to 20 mg/d and PTU up to 450 mg/d are considered safe during lactation.^59^

### Thyroid Eye Disease

TED is an autoimmune disease driven by TRAb activation of orbital fibroblasts resulting in extraocular muscle enlargement and orbital fat expansion. Its annual incidence is 16 cases in 100,000 women and 3 per 100,000 men.^60^ The most important risk factors for TED are smoking, older age group, high TRAb levels, high pretreatment levels of T3 and T4, and RAI therapy.^47^ Pathophysiology and treatment options are detailed elsewhere.^61,62^

In mild cases, medical therapy with topical cyclosporine and glucocorticoid ophthalmic drops may reduce ocular inflammation. Selenium (100 μg twice daily for 6 months) has been shown to improve eye manifestations and quality of life as well as slow the progression of mild disease, particularly in geographic areas where selenium deficiency is prevalent.^63^ In moderate to severe disease, initial medical treatment with high-dose glucocorticoids or teprotumumab is currently available.

Teprotumumab (Tepezza) is a monoclonal antibody that blocks the IGF-1 receptor, and was approved by U.S. Food and Drug Administration in 2020. Two 24-week trials compared teprotumumab with placebo in 171 patients between the 2 studies with active, moderate to severe orbitopathy.^64,65^ Both studies demonstrated that teprotumumab was superior to placebo in reduction of proptosis and clinical activity score (CAS). In the first trial by Smith et al^64^ in 2017, improvement of CAS by ≥2 points and reduction in proptosis by ≥2 mm, together, occurred in 69% of patients with teprotumumab versus 20% with placebo. In the second trial by Douglas et al^65^ in 2020, the primary outcome of proptosis improvement by ≥2 mm occurred in 83% of patients treated with teprotumumab versus 10% with placebo, whereas a secondary outcome of combined improvement in CAS and proptosis occurred in 78% with teprotumumab versus 7% with placebo. Teprotumumab is administered intravenously every 3 weeks (10 mg/kg first dose, then 20 mg/kg) for a total of 8 infusions. It is contraindicated in pregnancy and not approved for children below 18 years of age.

Other immunosuppressants like mycophenolate, tocilizumab, and rituximab have also been studied in the treatment of TED. Selection of secondary therapies should be individualized. Statins have been shown to prevent the development of TED^66^ and are also studied for its treatment. A phase 2 clinical trial conducted by Lanzolla et al^67^ demonstrated that adding atorvastatin 20 mg daily to IV glucocorticoids for 24 weeks improved overall clinical response (exophthalmos, CAS, eyelid aperture, and diplopia) and quality of life. The underlying mechanisms of statin effects in managing TED are not well understood, but one of the prominent hypotheses is the role of its pleiotropic anti-inflammatory effects.^68^ In many cases, however, surgery remains the necessary option for TED.

### Graves Dermopathy

Graves dermopathy usually does not need treatment. If treatment is considered, topical high potency glucocorticoid with occlusive dressing can be considered. Rituximab treatment to reduce B cells may be beneficial, but it remains experimental.

### Subclinical Hyperthyroidism

Subclinical hyperthyroidism (SCH) is defined with TSH levels below normal range with normal FT4 and FT3. Prevalence of SCH globally is 0.7% to 1.4%.^69,70^ SCH can progress to overt hyperthyroidism in 8% of patients by 1 year and 26% of patients by 5 year.^71^ In populations with sufficient iodine intake, GD is the cause of 40% of cases of SCH. Baseline serum level of TSH, not cause of disease, is the best predictor of progression of subclinical hyperthyroidism to overt hyperthyroidism.^72,73^

Treatment of SCH is recommended when serum thyrotropin levels are <0.1 mU/L, in persons aged >65 years and postmenopausal women due to association with adverse outcomes including cardiovascular disease, bone loss, fractures, and dementia.^72^ MMI remains an effective and safe choice for SCH due to GD.^74^ For asymptomatic individuals aged <65 years and a TSH level between 0.1 mIU/L and the lower limit of normal, observation is generally appropriate.

### Thyroid Storm

Thyroid storm is an acute, life-threatening complication of hyperthyroidism that presents with multisystem involvement and is associated with a significant mortality rate of 8% to 25%.^20^ Goals of treatment are to control increased adrenergic tone, achieve euthyroidism quickly and safely by reducing thyroid hormone synthesis, block thyroid hormone release, inhibit peripheral conversion of T4 to T3, and reduce enterohepatic recycling of thyroid hormone (Table 4).^20,75–77^

Although all ATDs decrease thyroid hormone synthesis, the current ATA guidelines recommend initiating PTU in patients with thyroid storm as it blocks T4 to T3 conversion. However, recent data suggest no difference in cost, mortality, and adverse events for those treated with PTU versus MMI.^78^

### Perioperative Preparation of Patients With GD for Thyroidectomy

Surgical intervention may be indicated in some patients with GD who have characteristics, including inability to tolerate ATD, refractory disease, very large goiter, concern for thyroid cancer, moderate to severe TED, or patient preference. Total thyroidectomy is generally more effective than subtotal thyroidectomy, with equal rates of complications, and is therefore preferred.^79^

Preparation for surgery is important to prevent thyroid storm. Thyroid gland is highly vascular and edematous under hyperthyroid state and hyperthyroidism is associated with metabolic dysfunction, tachycardia and heart failure. Hence, achieving euthyroidism prior to surgery is recommended. Perioperative management includes treatment with ATDs to decrease thyroid hormone production, beta-blockers to control the peripheral effects of excess free hormones, and iodine therapy. Treatment with Lugol iodine or potassium iodide decreases gland vascularity, thus decreasing intraoperative blood loss.^80–82^ Thionamides alone to achieve euthyroidism require 3 to 8 weeks of treatment. Different iodine regimens varying in dose and duration have been proposed. The widely studied duration is an average of 10 days of treatment with an iodide dose of 100 to 200 mg/d.^20,83^ In more emergent cases, treatment similar to that used during thyroid storm is useful (Table 4). In patients who are intolerant of ATDs, a regimen consisting of potassium iodide, beta-blockers, glucocorticoids, and cholestyramine should be used in the immediate preoperative period.^20,84^ Therapeutic plasma exchange is proposed as a secondline therapy due to the advantage of rapid fall in thyroid hormones, antibodies, cytokines, and catecholamines.^85,86^ Patients undergoing thyroidectomy are also at risk for postsurgical hypoparathyroidism and subsequent hypocalcemia due to accidental removal or devascularization of parathyroid glands during surgery. Studies have shown that preoperative vitamin D deficiency, especially levels <10 ng/mL, was associated with development of postsurgical hypocalcemia. Adequate vitamin D supplementation can prevent this complication.^87,88^

### Immune Checkpoint Inhibitor and Immunomodulatory Drug-Induced GD

Thyroid dysfunction is a rare but well-known side effect of immune checkpoint inhibitor (ICI) therapies, often associated with positive TPO antibodies.^89,90^ The clinical course is typically a brief period of hyperthyroidism that transitions to hypothyroidism requiring thyroid hormone replacement. ICI, to lesser extent, has been reported to cause GD.^91,92^ Some of these reports are limited in that TRAb status prior to receiving ICI was unknown. Offending agents include programmed cell death protein 1 inhibitors pembrolizumab^93^ and nivolumab,^94^ cytotoxic T-lymphocyte associated protein 4 (CTLA-4) inhibitors ipilimumab^95^ and tremelimumab.^96^

Cases of ipilimumab-associated GD where TED was the presenting manifestation with normal thyroid function tests^97^ and ipilimumab-induced thyroid storm in a patient with negative TSI and TRAb were reported.^98^ In most cases of ICI-induced GD, MMI was an effective treatment. Assessing for TRAb/TSI in addition to TPO Ab can be useful in ICI-induced hyperthyroidism if GD is suspected.

Alemtuzumab, a monoclonal antibody used effectively for multiple sclerosis, has been linked with development of GD; however, the mechanism remains unclear.^99^ GD has also been reported in several cases of HIV patients on high activity antiretroviral therapy.^100^

### Future Directions

Several new therapies for GD are currently under investigation. These new drugs can be grouped into 4 categories based on their mechanism of action: B lymphocyte immunomodulators (rituximab, iscalimab, and belimumab), TSH receptor antagonists, immunomodulatory TSH receptor peptides, and neonatal Fc receptor blockers that prevent immunoglobulin recycling (rozanolixizumab and efgartigimod). The results of small open label studies with these agents have been promising, but randomized multicenter trials are still to come.^101^ Addition of low-dose methotrexate 10 mg/wk to MMI was recently studied and resulted in a higher discontinuation rate as well as faster improvement of TRAb levels to homeostatic levels than MMI alone.^102^

## Conclusion

ATD, RAI, and thyroidectomy are 3 main treatment options when managing GD. Although RAI and surgery are associated with a high likelihood of subsequent hypothyroidism, ATD provide a way to achieve euthyroidism without increasing the risk for complications. ATDs have been the initial choice of treatment in many countries for years and have gained considerable popularity in the United States over the past 2 decades. The conventional duration of ATDs use for 18 to 24 as currently suggested by the ATA is associated with high relapse rates with GD. Long-term management with ATD for 5 years or more has demonstrated safety with higher rates of remission.

## Figures and Tables

**Table 1:** Risk Factors Associated With Graves Disease

Nonmodifiable^a^	Modifiable/environmental factors

Genetic predisposition: HLA-DR3, CTLA-4, TSH-R	Smoking
Estrogen exposure/female sex	Iodine exposure
Postpartum	Selenium deficiency
	Vitamin D deficiency
	Viral infections^b^
	Agent Orange exposure
	HCV-related mixed cryoglobulinemia
	Medications—ICI, alemtuzumab, HAART in HIV

Abbreviations: CTLA-4 = cytotoxic T-lymphocyte associated protein 4; HAART = highly active antiretroviral therapy; HLA = human leukocyte antigen; ICI = immune checkpoint inhibitors; TSH-R = thyroid-stimulating hormone receptor.

a Nonmodifiable risk factors are present in majority of patients.

b Viruses studied include Epstein-Barr virus, parvovirus-B19, foamy viruses, hepatitis C virus.

**Table 2:** Initial Medical Management of Graves Disease

Medication class	Dosage and frequency	Considerations	Side effects

Beta-blockers			
Propranolol	10–40 mg 3–4 times a day	Nonselective beta-blockade, preferred in pregnancy May block T4 to T3 conversion	Cardiac: heart failure exacerbation, bradycardia Noncardiac: bronchoconstriction, depression, fatigue, sexual dysfunction
Metoprolol	25–50 mg 2–3 times a day	Beta one selective	
Atenolol	25–100 mg 1–2 times a day	Once daily dosing, better compliance, avoid in pregnancy	
Esmolol	IV pump 50–100 μg/kg/min	ICU setting in severe thyrotoxicosis or storm	
Antithyroid medications			
Methimazole	5–40 mg daily^a^	First-line Better efficacy and safety, better compliance	Minor (5%): gastrointestinal distress and pruritis Major (<0.5%): agranulocytosis and hepatotoxicity (cholestatic or hepatocellular), vasculitis, pancreatitis
Propylthiouracil	50–150 mg 3 times a day	Second-line (if unable to tolerate methimazole) Preferred in first trimester of pregnancy	

ICU = intensive care unit.

a Higher doses of 30–40 mg/d may be required for patients with severe hyperthyroidism or larger goiter.

**Table 3:** Predictors of Response to Antithyroid Medications

Biochemically responsive/remission	Biochemically persistent/recurrence

• Higher frequency of hypothyroidism during treatment	Strong evidence:
	• TRAb >8.0 mIU/L and persistence
	• Goiter size/thyroid volume
• Lesser degree of thyrotoxicosis	• Smoking
• Smaller goiter	• Postpartum period
• TRAb <3.0 mIU/L	Possible/uncertain:
	• Higher maintenance dose of MMI
	• Graves ophthalmopathy at presentation
	• Higher FT4 levels
	• Insomnia
	• Male sex
	• Mental disorder
	• Use of iodized salt
	• Young age
	• Family history
	• GREAT score class II or GREAT+ score IV

Abbreviations: FT4 = free thyroxine; GREAT = Graves Recurrent Events After Therapy; MMI = methimazole; TRAb = thyrotropin receptor antibody.

**Table 4:** Management of Thyroid Storm

Treatment goals	Class	Medications	Dosing	Considerations

1. Control increased adrenergic tone	Beta-adrenergic receptor antagonists	Propranolol	40–80 mg every 4–6 h	
		Esmolol drip	Loading dose of 250–500 μg/kg followed by 50–100 μg/kg/min	
2. Reduce thyroid hormone synthesis	Thionamides	PTU	Loading dose of 500–1000 mg followed by 250 mg every 4 h	Blocks peripheral conversion of T4 to T3
		Methimazole	20 mg every 4–6 h	
3. Block release of thyroid hormone	Iodine preparations	SSKI	5 (250 mg or 0.25 mL) drops every 6 hours. Each drop contains 35–50 mg of iodine	Initiate 1 h after ATD to prevent imminent increase in thyroid hormone synthesis due to increased iodine load (Jod-Basedow phenomenon)
		Lugol solution/potassium iodide-iodine	5–7 drops every 6–8 h. Each drop contains ~6.25 mg of iodine per drop	
4. Inhibit conversion of T4 to T3	Glucocorticoids	Hydrocortisone	300 mg iv load followed by 100 mg every 8 h	Also provides prophylaxis against concomitant adrenal insufficiency
		Dexamethasone	0.1–0.2 mg/kg/d in divided doses every 6–8 h	
	PTU	Described above		
	Propranolol			
5. Reduce enterohepatic recycling of thyroid hormone	Bile acid sequestrants	Cholestyramine	4 g every 6 h	
		Colestipol-HCl	20–30 g daily	

Abbreviations: ATD = antithyroid drug; PTU = propylthiouracil; SSKI = supersaturated potassium iodine.

## Box

Usefulness of TRAb in Certain Scenarios

**Table:**

TRAb measurement can be useful in the following scenarios:

• Hyperthyroidism during pregnancy
• Patients with possible TED without biochemical hyperthyroidism
• Recent iodine load where thyroid uptake scan cannot be reliable, eg, recent amiodarone use, recent imaging studies with iodinated contrast
• To determine the prognosis for remission in those treated with ATD

Abbreviations: ATD = antithyroid drugs; TED = thyroid eye disease.

## Highlights

- Treatment of Graves disease include antithyroid drugs (ATDs), radioactive iodine, or surgery with increasing use of ATDs
- Relapse has been observed in >50% of patients treated with ATDs for the traditional 12- to 24-month duration
- Long-term ATD therapy has proven to be safe and effective, with significant improvement in quality of life
- Thyrotropin receptor antibody titers can help predict risk of recurrence
- Low doses of ATDs are safe in pregnancy and lactation

## Clinical Relevance

This article provides practical guidance for clinicians treating Graves disease (GD) with primary focus on medical therapy. GD accounts for 60% to 80% cases of hyperthyroidism and delay in treatment is associated with significant morbidity and mortality. This clinical review discussed strategies for medical management in GD including long-term use and monitoring of antithyroid drugs.

## Abbreviations:

ATA: American Thyroid Association
ATD: antithyroid drug
CAS: clinical activity score
FT4: free thyroxine
FT3: Free triiodothyronine
GD: Graves disease
GREAT: Graves Recurrent Events After Therapy
ICI: immune checkpoint inhibitor
IGF-1: insulin-like growth factor
MMI: methimazole
PTU: propylthiouracil
RAI: radioactive iodine
SCH: subclinical hyperthyroidism
TBI: thyrotropin-binding inhibiting immunoglobulin
TED: thyroid eye disease
TPO: thyroid peroxidase
TRAb: thyrotropin receptor antibody
TSH: thyroid-stimulating hormone
TSI: thyrotropin-stimulating immunoglobulin
